# Effect of the B:Zn:H_2_O Molar Ratio on the Properties of Poly(Vinyl Acetate) and Zinc Borate-Based Intumescent Coating Materials Exposed to a Quasi-Real Cellulosic Fire

**DOI:** 10.3390/polym12112542

**Published:** 2020-10-30

**Authors:** Jakub Łopiński, Beata Schmidt, Yongping Bai, Krzysztof Kowalczyk

**Affiliations:** 1 Faculty of Chemical Technology and Engineering, West Pomeranian University of Technology in Szczecin, Piastów Ave. 42, 71-065 Szczecin, Poland; jakub.lopinski@zut.edu.pl (J.Ł.); beata.schmidt@zut.edu.pl (B.S.); 2 School of Chemistry and Chemical Engineering, Harbin Institute of Technology, Harbin 150000, China; baifengbai@hit.edu.cn

**Keywords:** poly(vinyl acetate), intumescent coating, zinc borate, cellulosic fire, flame retardancy

## Abstract

In order to investigate an influence of the B:Zn:H_2_O molar ratio on the fire protection efficiency of poly(vinyl acetate)-based thermoplastic intumescent coating materials (ICs), systems containing ammonium polyphosphate, melamine, pentaerythritol and different types of zinc borates (ZBs) were tested in a vertical position in quasi-real fire conditions. 3ZnO·2B_2_O_3_·6H_2_O (ZB6), 2ZnO·3B_2_O_3_·3.5H_2_O (ZB3.5) or 3ZnO·2B_2_O_3_ (ZB0) were added in amounts of 1–10 wt. parts/100 wt. parts of the other coating components mixture. Char formation processes and thermal insulation features were investigated using an open-flame furnace heated according to the cellulosic fire curve. Thermogravimetric features (DTG), chemical structures (FTIR) and mechanical strength of the ICs and the chars were analyzed as well. It was revealed that the type and dose of the ZBs significantly affect thermal insulation time (TIT) (up to 450 °C of a steel substrate) and sagging (SI) of the fire-heated coatings as well as the compressive strength of the created chars. The highest TIT value (+89%) was noted for the sample with 2.5 wt. parts of ZB3.5 while the lowest SI (−65%) was observed for the coatings containing 10 wt. parts of the hydrated borates (i.e., ZB3.5 or ZB6). The best mechanical strength was registered for the sample filled with the anhydrous modifier (3 wt. parts of ZB0). The presented results show that the ICs with the proper ZBs can be used for effective fire protection of vertically positioned steel elements.

## 1. Introduction

Many types of active and passive fire protection of structures and buildings are known. They are intended to signal a fire, extinguish it, or—in the case of the passive safety measures—to provide more time for the emergency evacuation of people. The most important kind of the passive fire protection is covering the metallic construction elements of buildings by intumescent coatings (ICs) in order to reduce the destructive effect of a high temperature. Structural steels (depending on their composition) lose their load bearing capacity above the temperatures of 450–500 °C [1]. The main purpose of the ICs is prolongation the time needed to reach these critical temperature values by the steel substrate during a fire (i.e., the time before building collapse). Although the intumescent phenomenon has been known since the early 1970s [2], various ICs are still tested and developed. Different thermoplastics and reactive film-forming polymers are used as bases of the ICs, e.g., polyacrylates, phenoplasts, epoxides and polyurethanes. Except for the binder, the ICs consist of three main thermoactive components. Ammonium polyphosphate (APP) generates phosphoric acids (during its thermal degradation), which react with pentaerythritol (PER) and form an expanded carbon structure at a high temperature. At the temperature above 350 °C, melamine (MEL) releases non-flammable gases foaming the charred layer [3,4,5,6]. The char exhibits barrier and thermal insulation properties, thus it hinders mass (and heat) exchange between the environment and the covered steel substrate. Hot air masses are also accumulated in pores of the char, hence their size and distribution have a crucial influence on the thermal protection effectiveness of the ICs [7]. In order to improve the fire protection, various types of fillers and additives are incorporated into the coating systems as well. For example, titanium dioxide increases mechanical strength of the chars [8,9,10,11,12]. Additionally, researches pay the attention to systems with nanofillers [1,7,13,14], waste polymers [15] and bio-fillers [3,16,17,18]. In a few cases, boron compounds (mainly zinc borate such as 2ZnO·3B_2_O_3_·3.5H_2_O) have been used as flame retardants in ICs and as modifiers of the mechanical properties of charred layers [19,20,21]. It was stated that hydrated zinc borates dehydrate endothermically, they absorb heat from a polymeric material and the emitted water vapor dilutes flammable gaseous components [20]. Considering this thesis, it could be claimed that anhydrous zinc borates should not be used in the fire protective materials, however, there are no scientific publications describing an influence of the water content and the zinc borate type (i.e., the Zn and B concentration) on the char formation process and the mechanical properties of ICs. Unfortunately, the importance of the chemical composition of the hydrated boron compounds has not been determined in conditions even slightly similar to real or standardized fires. Generally, the Bunsen burner test is used for evaluation of the thermal insulation features of the ICs [20,21]. Nevertheless, due to a constant distance between the burner flame and a sample (and the constant temperature of the flame near to the tested IC), this method does not represent the fire with a determined temperature increment during a flashover period.

**Figure 1:**
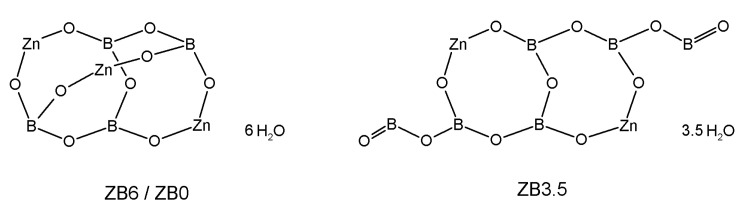
Structures of the tested zinc borates: 3ZnO·2B_2_O_3_·6H_2_O (ZB6), 2ZnO·3B_2_O_3_·3.5H_2_O (ZB3.5) and 3ZnO·2B_2_O_3_ (ZB0).

This article presents investigation results of a poly(vinyl acetate)-based intumescent coating material filled with various zinc borates (ZBs) and tested on a steel substrate in quasi-real fire conditions. Thermal degradation and mechanical features of the systems containing the anhydrous or hydrated ZBs (3.5H_2_O or 6H_2_O; Figure 1)—as well as APP, MEL, and PER mixtures—have been discussed in relation to their dose and Zn:B:H_2_O molar ratio. It is generally known that thermoplastic ICs often sag during a fire, thus char formation processes have been realized in a vertical position. The tests have revealed that only a proper concentration of a selected zinc borate in the poly(vinyl acetate)-based system results in good thermal and mechanical protection of the steel substrate exposed to a cellulosic fire.

## 2. Materials and Methods

### 2.1. Materials

The intumescent paints and coatings were prepared using the technical grade components:

- Poly(vinyl acetate) (PVAc) with an average molecular weight of 176,000 g/mol, glass transition temperature ca. 42 °C, softening temperature 150 °C and solid content >99% (M50; Synthomer, Essex, UK);
- Ammonium polyphosphate (type II) (APP), a powder with an average particles diameter of 18 µm (FR Cross 484; Budenheim, Germany);
- Melamine (MEL), a powder with a particles size ≤40 µm (Melafine; OCI Nitrogen, Geleen, The Netherlands);
- Pentaerythritol (PER), a powder with a particles size ≤15 µm (Charmor PM15; Perstorp Specialty Chemicals, Perstorp, Sweden);
- Titanium dioxide (rutile type TiO_2_) with an average particles size of 290 nm (Tytanpol R-001; GA Z.Ch. Police, Police, Poland);
- A wetting/dispersing additive based on a hydroxyl-functional carboxylic acid ester (Disperbyk 108; BYK-Chemie, Wesel, Germany);
- A silicone defoamer (Byk-066N; BYK-Chemie);
- Hydrated zinc borates: 3ZnO·2B_2_O_3_·6H_2_O (ZB6; POCh, Poland) and 2ZnO·3B_2_O_3_·3.5H_2_O (ZB3.5; Firebrake ZB, U.S. Borax, Chicago, IL, USA) with a particles size of ca. 20 µm;
- Anhydrous zinc borate (3ZnO·2B_2_O_3_) with a particles size of ca. 20 µm (ZB0, ZUT, Szczecin, Poland);
- n-Butyl acetate (Chempur, Piekary Śląskie, Poland).

### 2.2. Samples Preparation

PVAc was dissolved in n-butyl acetate and mixed (500 rpm, 5 min) with the auxiliary additives using the laboratory dissolver with a heavy-duty dispersion impeller (VMA Getzmann GmbH, Reichshof, Germany). Then, APP, MEL, PER, TiO_2_, and (optionally) the zinc borate were added to the system and homogenized at 3000 rpm for 1 h. The composition of the reference paint (IC-0) is specified in Table 1. The zinc borates (ZBs) were added in the amounts of 1–10 wt. parts/100 wt. parts of the reference paint solids (Table 2). The coating compositions (65% of solids) were applied by means of an adjustable gap applicator (Ascott, Tamworth, UK) onto a low-carbon steel substrate with the dimensions of 102 mm × 152 mm × 0.81 mm (Q-Panels, Q-Lab Europe, Bolton, UK). The coatings were dried at RT for 24 h and at 45 °C for 48 h. Subsequently, the samples were cut for panels with the dimensions of 100 mm × 100 mm; the thickness of the dry intumescent layers was 1 ± 0.03 mm. Samples of PVAc/ZB mixtures (for thermogravimetric tests) were prepared by mechanical mixing of the PVAc solution with ZB0 (37 wt. parts; the sample abbreviated to PVAc/ZB0), ZB3.5 (28 wt. parts; PVAc/ZB3.5) or ZB6 (48 wt. parts/100 wt. parts of PVAc; PVAc/ZB6). Then, the compositions were dried at RT (24 h) and at 45 °C for 48 h.

### 2.3. Methods

**Figure 2:**
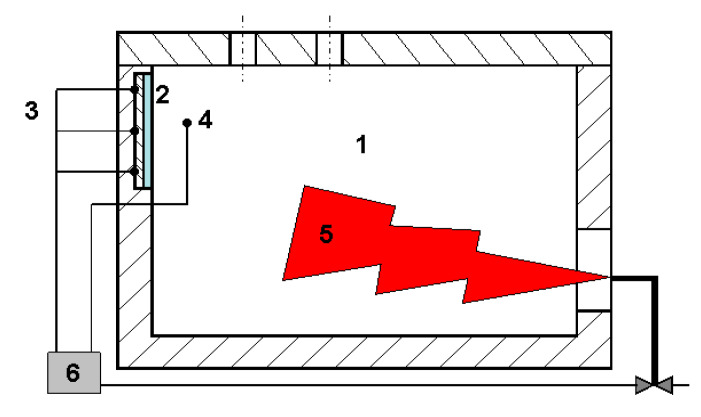
A scheme of the propane gas flame-heated programmable furnace used for the thermal insulation tests of the coatings: (1) the furnace chamber, (2) a steel substrate with the intumescent coating, (3) three thermocouples measuring temperature of the steel substrate, (4) a thermocouple measuring temperature of the furnace chamber, (5) a propane gas burner and (6) a temperature control unit.

The thickness of the dry intumescent coatings was measured with the electronic film gauge (Byko-test 8500; BYK-Gardner, Geretsried, Germany). Fire characteristics of the coatings (applied onto the steel plates) were investigated by means of a programmable laboratory furnace directly heated by an open flame of propane gas (Figure 2). The temperature in a furnace chamber (*T_F_*) was automatically regulated (acc. to the standard cellulosic fire curve described in PN-EN 1991-1-2:2006) by a control unit of a gas burner:

(1) $$T_{F}=345\;\log\left(8t+1\right)+20\left({}^{\circ}C\right)$$

where: *t*—time of the test [min].

The *T_F_* and temperature of the steel substrates were monitored using K-type thermocouples and a PC unit. Thermal insulation time (TIT) was presented as a time needed to reach the temperature of 450 °C (the critical temperature value) measured on a backside of the tested sample.

The thickness of charred samples (i.e., after the furnace test) was determined using a dial gauge with a stand (five measurements for the each sample). The intumescent index (*INI*) for the coatings was calculated according to the equation [15]:

(2) $$INI=\frac{L_{1}-L}{L_{o}}\left(a.u.\right)$$

where: *L* is the thickness of the steel substrate (i.e., 0.81 mm), *L*_0_ is a thickness of a dry coating before the furnace test (mm) and *L*_1_ is a thickness of a char after the test (mm).

The sagging index (SI) represents sagging of the coatings during the furnace test (i.e., percentage of the steel substrate not covered by the charred layer) and was determined by a digital analysis of the sample image. Thermal analysis of the intumescent coatings and their components was carried out using the thermogravimetric analyzer (50 °C–900 °C, a heating rate of 100 °C/min, air atmosphere; TG Q5000, TA Instruments, New Castle, DE, USA). Selected chars were also milled and investigated using the Fourier Transform Infrared Spectroscope (FTIR) with attenuated total reflectance (ATR) accessories (Thermo Nicolet Nexus FT-IR, Thermo Electron, Waltham, MA, USA). The compressive strength of the charred intumescent coatings was monitored using the Z010 machine (Zwick/Roell, Ulm, Germany) equipped with a flat steel indenter with a diameter of 20 mm (indentation speed of 10 mm/min). Compressive tension values at the strain of 5%, 15%, and 25% were calculated by means of the TestXpert II software (Zwick/Roell).

## 3. Results and Discussion

### 3.1. Furnace Test Results

**Figure 3:**
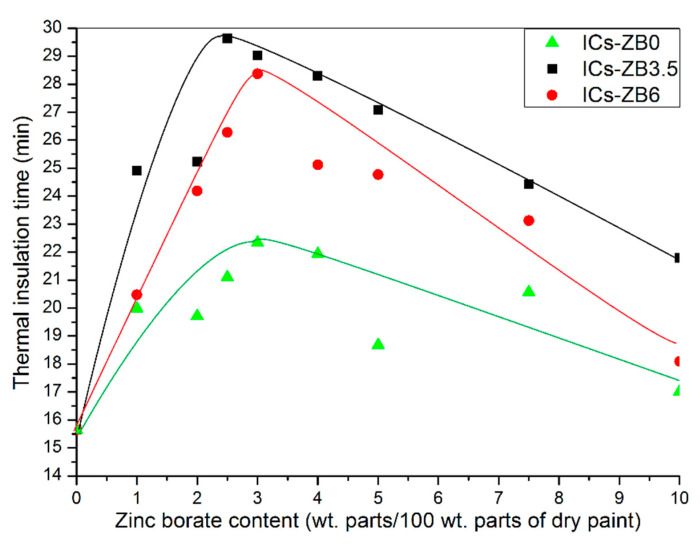
Thermal insulation time for the intumescent coatings as a function of zinc borate content.

**Figure 4:**
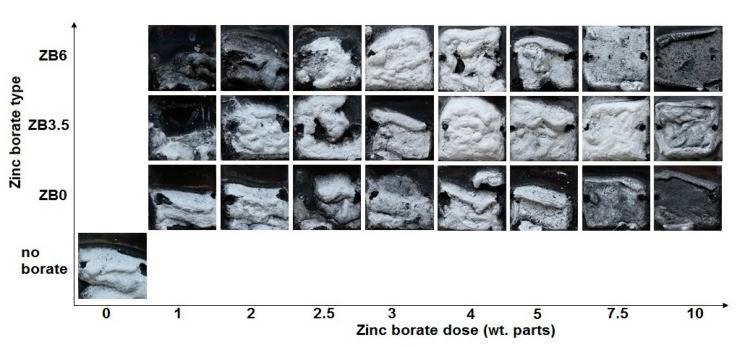
Digital images of the charred intumescent coatings (after the furnace test) with various zinc borates.

**Figure 5:**
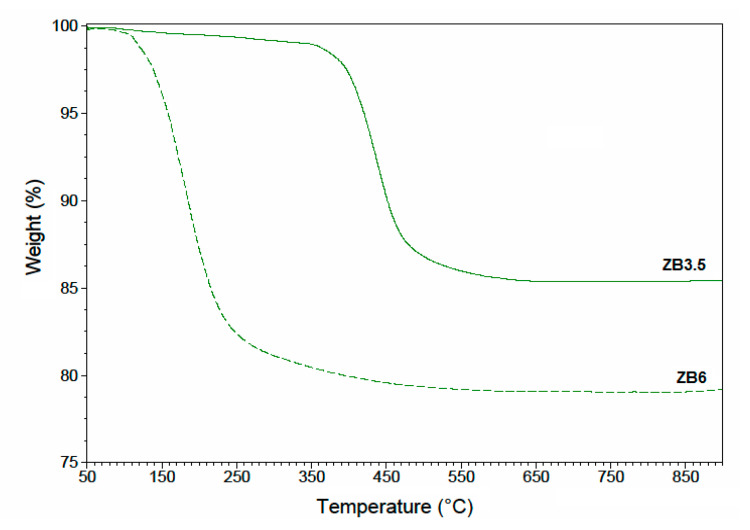
Thermogravimetric curves for the hydrated zinc borates.

**Figure 6:**
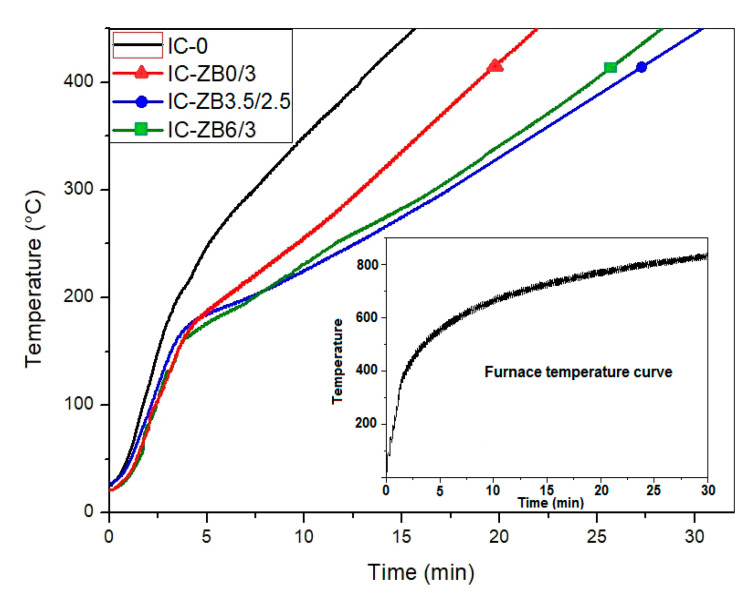
Thermal insulation curves for the selected intumescent coatings with various zinc borates.

Results of the fire resistance test of the poly(vinyl acetate)-based intumescent coatings are presented in Figure 3, while digital images of the samples (after the furnace test) are shown in Figure 4. In detail, the curves represent a relation between thermal insulation time values (TIT) and the content of the zinc borates in the ICs. As can be seen, the incorporation of 1–10 wt. parts of the anhydrous (ZB0) or hydrated borates (ZB3.5 or ZB6) into the coating systems caused positive changes of TIT; even the lowest concentration of the ZBs (1 wt. part) resulted in a significant improvement of the analyzed parameter. In the case of the IC-ZB0/1 and IC-ZB6/1 samples, TIT was increased by 27% (20.0 min) and 30% (20.5 min; Table 2) while IC-ZB3.5/1 reached ca. 58% higher TIT value (24.9 min) in comparison to the reference coating without borates (15.7 min; IC-0). Nevertheless, a further TIT improvement was recorded for the systems with the higher ZB content as well. Interestingly, the highest TIT values for all the ZB-modified systems were reached at the similar contents of the borates, i.e., at 3 wt. parts of ZB0 (IC-ZB0/3), 2.5 wt. parts of ZB3.5 (IC-ZB3.5/2.5), and 3 wt. parts of ZB6 (IC-ZB6/3), but the recorded TIT values for these samples were markedly different (22.3 min for IC-ZB0/3, 29.6 min for IC-ZB3.5/2.5, and 28.4 min for IC-ZB6/3). The observed results for the hydrated ZBs-based coatings (i.e., the higher TIT values than for IC-ZB0/3 and IC-0) could be explained by an endothermic dehydration reaction of ZB3.5 [22] and ZB6 during the fire test; it is generally known that release of the crystalline water causes reduction of the heated system temperature. It is noteworthy that the thermogravimetric analyses of the both borates revealed a higher dehydration process temperature of ZB3.5 (maximal weight loss rate at 437 °C) in comparison to ZB6 (185 °C; Figure 5). Additionally, the former borate contains only 14.6 wt% of the water while its concentration in ZB6 is much higher (ca. 20.8 wt%). Considering shapes of the thermal insulation curves for IC-ZB3.5/2.5 and IC-ZB6/3—registered at the beginning of the furnace test of the ICs (Figure 6)—it can be claimed that the latter sample exhibited better thermal insulation features than IC-ZB3.5/2.5 and the other systems. In detail, after 5 min of their heating (i.e., at the temperature of the furnace ca. 580 °C), the steel substrate protected by IC-ZB6/3 reached lower temperature (176 °C) in relation to IC-ZB3.5/2.5 (185 °C), IC-ZB0/3 (187 °C), and the reference coating (247 °C; IC-0). It was caused by the mentioned low-temperature dehydrating process of ZB6 in the IC-ZB6/3 sample. Nevertheless, during the further heating of the samples (>8 min of the fire test), the temperature of the steel plate with IC-ZB3.5/2.5 was markedly decreased in comparison with IC-ZB6/3; it was resulted by the dehydration process of ZB3.5, which begins at the much higher temperature than for ZB6 (Figure 5). Finally, IC-ZB3.5/2.5 reached the higher TIT value (+1.2 min) than IC-ZB6/3 and the other samples. It shows that the dehydration process temperature is more important than the crystalline water content in ZB3.5 and ZB6, and it crucially affects the TIT parameter of the tested ICs with these borates.

Interestingly, the TIT values for the ZB0-based coatings were also significantly higher in relation to the reference sample, however, this borate does not contain the crystalline water. It should be pointed out that the higher thermal insulation efficiency of all the compositions with the anhydrous (or hydrated) ZBs could be affected by a crosslinking process of the polymeric coating binder by the borates [7]. Arguably, the PVAc-ZB interactions increase viscosity of the coatings (during the fire test) and affect formation processes of foamed layers. Thus, different shapes of the charred samples after the furnace test have been observed (Figure 4). Indeed, the carbonaceous layers exhibited varied intumescent index (INI), but also different sagging index values (SI; Table 2). The latter parameter describes denudation efficiency of a steel plate covered by a coating (i.e., its thermoplastic behavior) during its intensive fire heating. In the case of a high SI value, the tested steel sample is only partially protected by a char, thus its averaged TIT value is relatively low. In authors’ opinion, the sagging tendency of the ICs during a fire is a crucial feature of thermoplastic binder-based systems utilized at vertical positions. Therefore, taking into consideration the INI calculation methodology, this parameter (i.e., an increment of the coating thickness) is not sufficient to reliable description of intumescent efficiency of the tested coatings. For a better presentation of the ICs foamability (an increment of layer volume), the new parameter called normalized intumescent index (NINI) should be used. It was calculated according to the following equation:

(3) $$NINI=\frac{INI\cdot (100-SI)}{100}\left(a.u.\right)$$

**Scheme 1:**
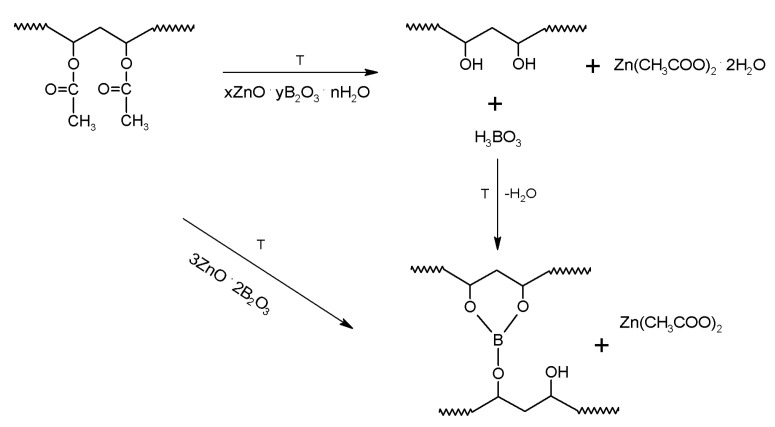
A scheme of PVAc crosslinking process by hydrated and anhydrous zinc borates.

**Figure 7:**
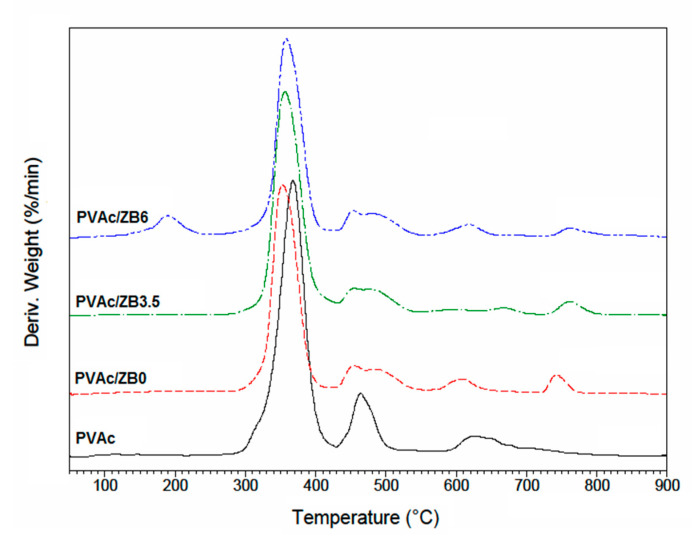
Thermogravimetric curves (the first derivative; DTG) for PVAc, PVAc/ZB0, PVAc/ZB3.5 and PVAc/ZB6.

As can be seen, the reference coating (IC-0) exhibited the relatively high SI value (28%; Table 2) and it noticeably increased up to 35, 55 or 48% after the addition of 1 wt. part of ZB0, ZB3.5 or ZB6, respectively. The INI values were also increased (from 27 to 32, 39 or 36 a.u., respectively) while NINI was relatively unchanged (18–21 a.u.). Nevertheless, higher doses of the borates (2–5 wt. parts; mainly the hydrated forms) caused significant limitation of the SI parameter (up to 12%) and only a slight increment of NINI (18–24 a.u.). It means that the ZBs addition (up to 5 wt. parts) reduces sagging of the coatings but does not markedly change volume of the charred layers. On the other hand, at the higher doses of the ZBs (>5 wt. parts), the values of the mentioned parameters (INI, SI, NINI) were diminished. Considering the recorded SI variations (resulted by the ZB type and content), it could be claimed that this phenomenon is simultaneously affected by the supposed crosslinking reaction of the PVAc-ZB system and—optionally for the ICs with ZB3.5 or ZB6—by the dehydration processes of the applied borates. Thus, it seems that the chemical composition of the tested borates is a crucial parameter affecting the analyzed features of the ICs. As mentioned above, ZB0 contains no water, therefore, the B and Zn content in this borate (11.3 wt% and 51.1 wt%, respectively) is higher in relation to ZB6. Nevertheless, the B:Zn molar ratio is the same for ZB0 and ZB6 (1:0.75; Table 3). On the other side, ZB6 consists the higher amount of the crystalline water and Zn (39.9 wt%) as well as much less B (8.8 wt%) in comparison with ZB3.5 (30.1 wt% of Zn, 14.9 wt% of B). As can be seen in Figure 4 (Table 2), the charred samples with ZB6 (>1 wt. part) exhibited generally lower sagging index values (21–15%) than the coatings filled with the anhydrous form of this additive (ZB0; 30–17%). This shows that the SI limitation was not directly caused by the B (or Zn) concentration in the coating, because ZB6 contains the lower amounts of these elements than ZB0. Additionally, it should be noted that the samples containing almost theoretical stoichiometric ratios of PVAc and these ZBs (needed to complete crosslinking of the polymer by boron [7]) reached also significantly different values of SI, i.e., 22% for IC-ZB0/5 and 10% for IC-ZB6/7.5 (Table 2). Thus, it can indicate that the water (released via the dehydration process of ZB6) positively affects the PVAc crosslinking phenomenon, e.g., by the facile hydrolysis of acetate groups and generation of poly(vinyl alcohol) (PVAl) or polyenes [23], boric acid (or other borate hydroxides) as well as zinc acetate dihydrate (as a basic form of this compound) [7,15] (Figure 7). According to the literature, boric acid needs cations (zinc) to be able to crosslink the PVAl matrix [24]. Perhaps their creation is also more effective in the ICs containing the crystalline water released from the hydrated zinc borates. On the other hand, it should be explained why the anhydrous borate (ZB0) reduces sagging of the PVAc-based coatings. In authors’ opinion, the water generated during dehydroxylation of pentaerythritol acts similar to the crystalline water generated by the hydrated borates. Due to the relatively high temperature of the former process (ca. 320 °C [7]), the sagging effect of the ZB0-based systems was relatively more effective in relation to the coatings filled with ZB6. Other explanation of the SI reduction observed for the ICs-ZB0-type coatings is a direct interchange reaction between PVAc and ZB0 (transesterification reaction, Scheme 1); in this case, the water would not be needed in the crosslinking process of the polymeric binder.

**Figure 8:**
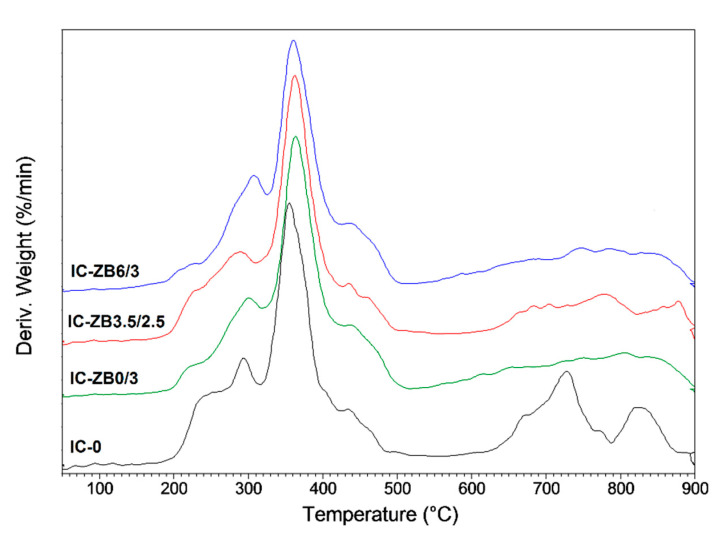
DTG curves for the selected intumescent coatings with various zinc borates.

It is noteworthy that the thermogravimetric analysis of the PVAc/ZB6 composition (i.e., the binder mixed with ZB6) revealed a significant weight loss of the sample at the temperature range of 140–235 °C (the maximal weight loss at 190 °C; Figure 7). The similar phenomenon was not recorded for PVAc/ZB0. The mentioned weight loss value correlates with the crystalline water content in ZB6. It indicates that the entire water evaporates from IC-ZB6-type systems at the relatively low temperature and before the main foaming process. Considering the hypothesis of the acetate groups hydrolysis and generation of poly(vinyl alcohol)/polyenes, boric acid/boron hydoxides, and zinc acetate dihydrate in the presence of the released crystalline water (Scheme 1), the crosslinking process of the binder and dehydration of zinc acetate dihydrate should be realized below 235 °C. In this case, the released water amount would be similar to the weight loss value recorded for the PVAc/ZB6 sample (according to [25], the dehydration process of zinc acetate dihydrate is realized at 103 °C). The presented hypothesis seems so be also right, because—as mentioned above—ICs with ZB6 (>1 wt. part) exhibited significantly lower SI values than IC/ZB0-type samples. It may confirm that the ICs with the hydrated borate (ZB6) were crosslinked at a lower temperature (the lower SI and the higher TIT values) than these with ZB0 (the higher SI and the lower TIT values). Considering the theory of the PVAc-ZB0 transesterification reaction (Scheme 1), the crystalline water would not be needed to realize the crosslinking process of the polymeric binder; finally, the chemical structures created from the ZB0 or ZB6-based systems should be similar. In this case, thermal stability of the systems should be similar as well. As can be seen in Figure 8, the DTG curves for IC-ZB0/3 and IC-ZB6/3 (or PVAc-ZB0 and PVAc-ZB6, Figure 7) are similar except for the peak at ca. 200 °C, which represents evaporation of the crystalline water from the ZB6-based coating. Additionally, the curves differ from these registered for IC-0 and IC-ZB3.5/2.5. It can represent that thermal degradation processes occurring during heating the IC-ZB0/3 and IC-ZB6/3 systems (i.e., the processes causing the weight loss of the samples) are similar and the crystalline water is not necessary for the PVAc-ZB crosslinking reactions (but positively affects them). Unfortunately, the water evaporation from the ZB3.5-based coatings was not revealed by the DTG analysis (Figure 7) due to similar temperatures of the zinc borate dehydration (439 °C, Figure 5) and the PVAc degradation processes. It should be noted that the crystalline water (before its evaporation) arguably plasticizes the binder of the IC-ZB6-type compositions, thus the SI value for the system with the lowest ZB6 dose (i.e., 48% for IC-ZB6/1) was markedly higher in comparison with IC-ZB0/1 (35%) and IC-0 (28%). That phenomenon resulted in a decrement of coating viscosity during the fire heating test (the similar process was observed for IC-ZB3.5/1; Table 2). Nevertheless, the higher doses of ZB6 or ZB3.5 significantly reduce the plasticizing effect (by more effective crosslinking of the binder); finally, it causes marked limitation of the SI parameter.

**Figure 9:**
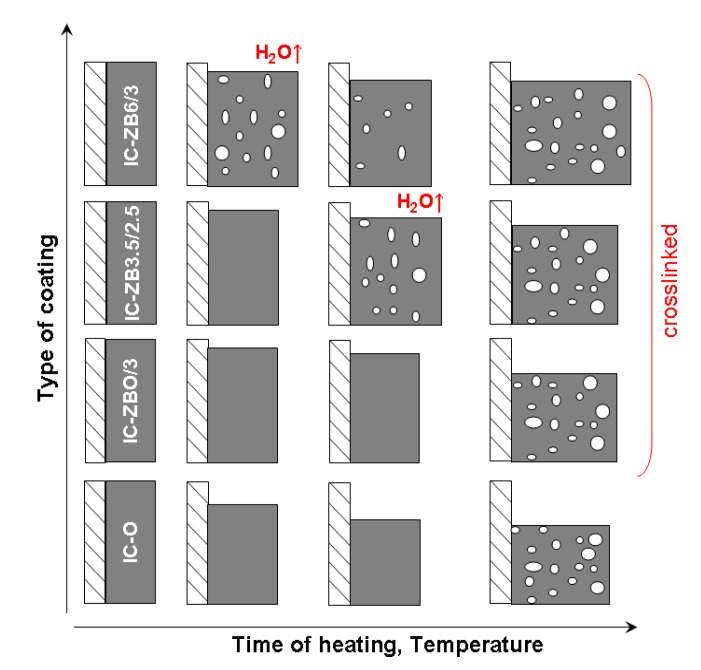
A schematic illustration of foaming process of the selected intumescent coatings with various zinc borates.

Interestingly, the NINI values calculated for the coatings modified with the anhydrous (ZB0) or hydrated zinc borates (ZB3.5 and ZB6) are quite comparable and vary in the range of 2–23 a.u. for IC-ZB0, 10–23 a.u. for IC-ZB3.5, and 3–24 a.u. for IC-ZB6 (Table 2). Especially, the samples containing the ZBs with the same Zn-B ratios (i.e., the IC-ZB0 and IC-ZB6-type systems) exhibit the similar ranges of the SI parameter values. It may confirm that the crystalline water (after its release from the hydrated borate) only slightly increases foamability of the coatings. Arguably, a major part of the water evaporates from the samples before the final char forming process. A scheme of charred coatings creation from IC-0, IC-ZB0/3, IC-ZB3.5/2.5, and IC-ZB6/3 systems (considering the crystalline water evaporation process and the registered SI and NINI parameters values) is presented in Figure 9.

### 3.2. Mechanical Features of the Chars

**Figure 10:**
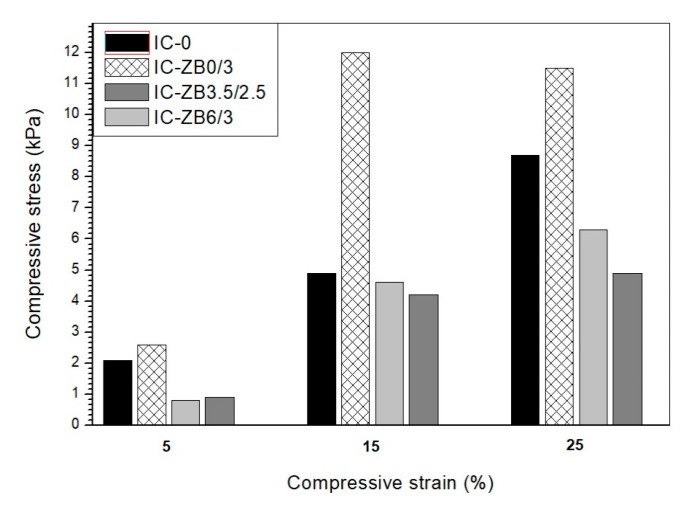
Compressive stress (at the selected strain) for the charred intumescent coatings with various zinc borates.

**Figure 11:**
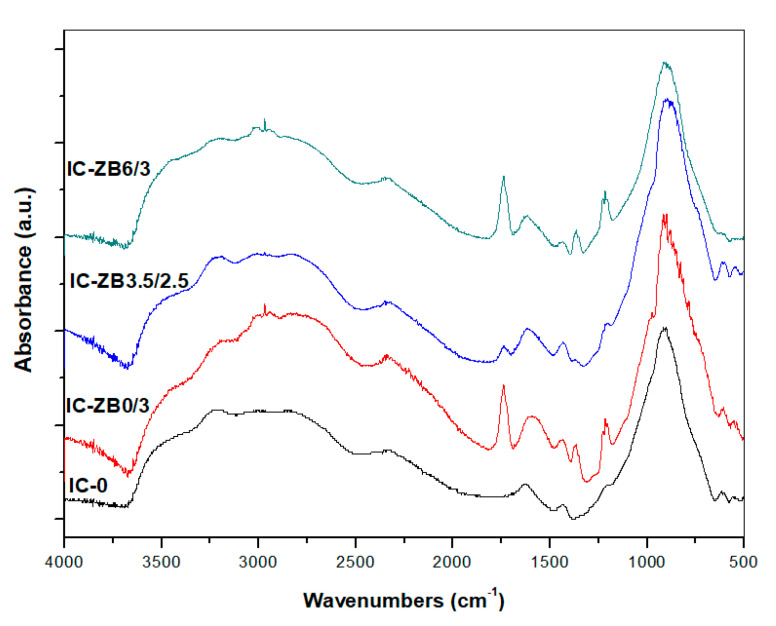
FTIR spectra for the charred intumescent coatings with various zinc borates.

The compressive strength values for the charred intumescent coatings (at the strain of 5, 15, and 25%) are presented in Figure 10. As can be seen, the introduction of the anhydrous zinc borate (ZB0) resulted in a significantly higher compression resistance, especially at the higher strain (12.0 kPa at 15% and 11.5 kPa at 25%) in comparison with the reference sample (4.9 and 8.7 kPa, respectively). Interestingly, the latter sample reached a higher mechanical strength than the other systems (4.6 and 6.3 kPa for IC-ZB3.5/2.5, 4.2 and 4.9 kPa for IC-ZB6/3). Taking into consideration the INI, SI, and NINI values for the investigated samples (Table 2), it could be claimed that these parameters do not correlate with the recorded mechanical features. Additionally, the compressive strength does not depend on the boron content in the samples (the highest concentration in IC-ZB3.5/2.5, the lowest in IC-ZB6/3). Nevertheless, it seems that the TIT parameter may influence the mechanical resistance of the charred layers due to the shorter heating time of the reference sample (15.7 min) and the sample with ZB0 (22.3 min) in comparison with IC-ZB3.5/2.5 (29.6 min) and IC-ZB6/3 (28.4 min; Table 2). In this case, the longer heating time to the critical temperature of the covered steel substrate (450 °C) negatively affected the compressive strength of the created char. On the other hand, IC-0 (the shorter heating time) exhibited markedly lower mechanical resistance than IC-ZB0/3 (the longer heating time). Arguably, the Zn presence in the latter sample may be considered as an additional factor affecting the feature of this material; IC-ZB0/3 contained the higher amount of Zn (ca. 1.5 wt%) in relation to the reference sample (no Zn), IC-ZB6/3 (ca. 1.2 wt%), and IC-ZB3.5/2.5 (ca. 0.7 wt%). As can be observed in Figure 11, the FTIR spectrum for the charred IC-ZB0/3 layer is similar to the IC-ZB6/3 spectrum except for the bands at ca. 970, 620, and 560 cm^−1^ (markedly registered for the former system). The band at 970 cm^−1^ is not significantly visible in the spectra for the charred reference sample and IC-ZB3.5/2.5. According to [26], the 623 and 566 cm^−1^ peaks represent O-P-O bonds while 961 cm^−1^ corresponds to P-O-P; they refer to the chemical structure of titanium pyrophosphate (TiP_2_O_7_), which improves mechanical features of charred intumescent coatings [12]. It means that charred IC-ZB0/3 contains much more TiP_2_O_7_ than the other samples, thus its mechanical strength is better. Probably, Zn catalyzes the TiP_2_O_7_ formation process via a reaction of TiO_2_ and APP. Nevertheless, it seems that the charred IC-ZB0/3 (and IC-ZB6/3) sample contains a zinc phosphate glass (ZnO-P_2_O_5_) as well. The characteristic bands for this compound (i.e., ca. 1380 cm^−1^ and 1220 cm^−1^ [27]) were markedly observed for the chars due to the relatively highest Zn concentration in these systems.

Considering the DTG curves for the tested coatings (Figure 8), it could be noted that the ZBs significantly affect their thermal stability at the temperature range of 650 °C–850 °C; the main changes of the DTG curves (vs. IC-0) were registered for the samples containing ZB0 and ZB6. According to [7], the weight loss at the mentioned temperatures represents thermal destruction processes of APP (or its derivatives). It seems that ZB0 and ZB6 markedly influence that phenomenon (in a similar way) due to the mentioned high Zn content in these borates.

The charred IC-ZB0/3 and IC-ZB6/3 layers consisted of detectable amounts of the zinc phosphate glass (ZnO-P_2_O_5_), however, that compound did not positively affect the compressive strength of the samples (the values of the mechanical parameter were different for IC-ZB0/3 and IC-ZB6/3, and similar for IC-ZB6/3 and IC-ZB3.5/2.5; Figure 10). In this case, the highest mechanical strength of ZB0/3 was resulted by the highest TiP_2_O_7_ content in this charred sample.

## 4. Conclusions

Considering the presented features of the PVAc-based intumescent coatings (ICs) modified with the various zinc borates (ZBs), it can be claimed that the anhydrous (ZB0) and hydrated ZBs (i.e., ZB3.5 with 3.5H_2_0, ZB6 with 6H_2_O/per molecule of the ZBs) positively affect the charring processes and thermal insulation time (TIT) of the fire-protective materials. The longest TIT was recorded for the sample filled with 2.5 wt. parts of ZB3.5/100 wt. parts of the other coating components, i.e., +13.9 min in relation to the reference sample (IC-0). It shows that the borate with the higher dehydration temperature (ZB3.5) more effectively elongates the TIT parameter than the ZB6 modifier (with the higher crystalline water content). The tested ZBs significantly reduce sagging of the formed chars (from 28% for IC-0 to 10% for the samples with 7.5 wt. parts of either ZB3.5 or ZB6), however, the ZB6 borate more effectively decreases this parameter than its anhydrous form (ZB0). It seems that the sagging of the chars is limited by the crosslinking processes of the poly(vinyl acetate) binder during its thermal degradation. The ZBs slightly increase foamability of the chars as well; the highest volumetric increment of the ICs during the fire tests (24 times vs. 19 times for IC-0) was registered for the sample filled with ZB6. That parameter reached 23 a.u. for the coatings with ZB0 or ZB3.5. Moreover, the anhydrous borate (ZB0) markedly increases the compressive strength of the formed chars (from 4.9 kPa to 12.0 kPa for the compressive strain of 15%). It was affected by the relatively higher TiP_2_O_7_ content in the ZB0-based charred layers in relation to the reference sample and the samples with the other borates.

## Figures and Tables

**Table 1:** The reference intumescent system composition.

Component	The Component Dose (wt. parts)
PVAc	16.0 ^1^
APP	40.9
MEL	20.4
PER	13.6
TiO_2_	5.0
Disperbyk 108	4.0
Byk 066N	0.1

^1^ Solid content of a solution in n-butyl acetate (wt. parts).

**Table 2:** Results of the furnace test of the intumescent coatings with various zinc borates.

Symbol	Zinc Borate Type	Zinc Borate Content (wt. parts) ^1^	TIT ^2^ (min)	INI ^3^ (a.u.)	SI ^4^ (%)	NINI ^5^ (a.u.)
IC-0	-	0	15.7	27	28	19
IC-ZB0/1	ZB0	1	20.0	32	35	21
IC-ZB0/2	ZB0	2	19.7	26	30	18
IC-ZB0/2.5	ZB0	2.5	21.1	30	23	23
IC-ZB0/3	ZB0	3	22.3	26	21	21
IC-ZB0/4	ZB0	4	21.9	25	19	20
IC-ZB0/5	ZB0	5	18.7	23	22	18
IC-ZB0/7.5	ZB0	7.5	20.6	13	20	10
IC-ZB0/10	ZB0	10	17.0	2	17	2
IC-ZB3.5/1	ZB3.5	1	24.9	39	55	18
IC-ZB3.5/2	ZB3.5	2	25.2	25	24	19
IC-ZB3.5/2.5	ZB3.5	2.5	29.6	26	19	21
IC-ZB3.5/3	ZB3.5	3	29.0	24	20	19
IC-ZB3.5/4	ZB3.5	4	28.3	26	13	23
IC-ZB3.5/5	ZB3.5	5	27.1	23	12	20
IC-ZB3.5/7.5	ZB3.5	7.5	24.4	18	10	16
IC-ZB3.5/10	ZB3.5	10	21.8	12	14	10
IC-ZB6/1	ZB6	1	20.5	36	48	19
IC-ZB6/2	ZB6	2	24.2	25	21	20
IC-ZB6/2.5	ZB6	2.5	26.3	27	17	22
IC-ZB6/3	ZB6	3	28.4	29	16	24
IC-ZB6/4	ZB6	4	25.1	26	18	21
IC-ZB6/5	ZB6	5	24.8	27	16	23
IC-ZB6/7.5	ZB6	7.5	23.1	16	10	14
IC-ZB6/10	ZB6	10	18.1	4	15	3

^1^ wt. parts of a zinc borate/100 wt. parts of the reference paint solids; ^2^ thermal insulation time; ^3^ intumescent index; ^4^ sagging index; ^5^ normalized intumescent index.

**Table 3:** Characteristics of the zinc borates.

Zinc Borate Symbol	Molecular Weight (g/mol)	Chemical Composition (wt%)			B:Zn:H_2_O Molar Ratio	PVAc:ZB Weight Ratio ^1^
		B	Zn	H_2_O		
ZB0	383.5	11.3	51.1	0	1:0.75:0	100:37
ZB3.5	434.7	14.9	30.1	14.5	1:0.33:0.58	100:28
ZB6	491.5	8.8	39.9	21.9	1:0.75:1.5	100:48

^1^ in relation to the stoichiometric ratio of VAc: B needed to complete crosslinking of PVAc.
